# Supplementary material for: Metabolomic Analysis Reveals Changes in Preimplantation Embryos Following Fresh or Vitrified Transfer
Source: Int J Mol Sci. 2020 Sep 26;21(19):7116. doi: 10.3390/ijms21197116 (PMC7582512; doi:10.3390/ijms21197116)
Supplement: Supplementary file 1 [file ijms-21-07116-s001.pdf]

Supplementary Table 1. Targeted identification of differentially accumulated metabolites in day-6 embryos after in vivo development by natural conception (NC) or following fresh (FT) or vitrified (VT) embryo transfer. Red denotes statistical differences at $p \leq 0.05$.

Metabolite	FT/NC			VT/NC			VT/FT		
	Fold change	p-value	adj p-value	Fold change	p-value	adj p-value	Fold change	p-value	adj p-value
**Arginine**	0,05	0,90	0,98	0,17	0,68	0,78	0,12	0,76	0,92
**Aspartate**	0,14	0,63	0,96	0,28	0,33	0,59	0,14	0,58	0,92
**Citrate**	-0,94	0,23	0,81	-0,79	0,31	0,59	0,15	0,88	0,92
**Gluconate**	-0,02	0,98	0,98	0,22	0,71	0,78	0,24	0,67	0,92
**Glucose**	1,04	0,39	0,81	1,53	0,16	0,45	0,49	0,49	0,92
**Glutamic_acid**	-0,47	0,20	0,81	-0,39	0,30	0,59	0,08	0,85	0,92
**Glutamine**	-0,99	**0,05**	0,37	-1,43	**0,03**	0,20	-0,43	0,60	0,92
**Glyceraldehyde-3P**	-0,44	0,60	0,96	-0,14	0,40	0,60	0,30	0,40	0,92
**Glycerone-P**	-0,47	0,36	0,81	-0,13	0,79	0,82	0,34	0,53	0,92
**Histidine**	0,01	0,98	0,98	0,27	0,51	0,66	0,26	0,51	0,92
**Isoleucine-leucine**	0,51	0,72	0,98	1,43	0,20	0,52	0,91	0,31	0,92
**Lysine**	-0,02	0,96	0,98	0,38	0,33	0,59	0,40	0,29	0,92
**Malate**	-0,44	0,49	0,94	-0,63	0,38	0,60	-0,19	0,81	0,92
**Methionine**	1,02	0,39	0,81	0,93	0,47	0,63	-0,09	0,92	0,92
**Phenylalanine**	-0,04	0,97	0,98	0,85	0,42	0,60	0,90	0,38	0,92
**Threonine**	-1,19	**0,04**	0,37	-1,10	**0,05**	0,27	0,08	0,92	0,92
**Tyrosine**	0,12	0,92	0,98	0,56	0,61	0,74	0,44	0,66	0,92
**ß-D-Glucose-6P**	0,72	0,32	0,81	1,14	0,09	0,31	0,42	0,39	0,92
**Succinate**	-0,86	**0,03**	0,37	-1,13	**0,02**	0,20	-0,27	0,62	0,92
**Serine**	-0,02	0,96	0,98	0,10	0,83	0,83	0,12	0,79	0,92
**Proline**	-0,20	0,31	0,81	-0,54	**0,04**	0,20	-0,34	0,17	0,92
**Valine**	0,35	0,61	0,96	1,30	**0,03**	0,20	0,95	**0,05**	0,92
**11,14,15-theta**	-1,60	0,07	0,33	-2,00	0,06	0,23	-0,40	0,82	1,00

**5,6-Dihydroxy-8Z,11Z,14Z-eicosatrienoic_acid**	-0,65	0,28	0,52	-1,04	0,16	0,29	-0,39	0,65	1,00
**5-hete**	-1,98	**0,02**	0,29	-2,95	**0,01**	0,22	-0,97	0,67	1,00
**6-ketoprostaglandin_e1**	-0,34	0,64	0,77	-0,26	0,73	0,81	0,08	0,92	1,00
**Adrenic_acid**	-3,04	**0,05**	0,29	-4,90	**0,05**	0,23	-1,86	0,83	1,00
**Arachidic_acid**	-1,25	0,10	0,33	-1,63	0,07	0,23	-0,38	0,76	1,00
**Arachidonic_acid**	-3,00	**0,05**	0,29	-4,43	**0,05**	0,23	-1,43	0,85	1,00
**Behenic_acid**	-0,08	0,90	0,92	-0,02	0,98	0,98	0,06	0,93	1,00
**Docosadienoic_acid**	-1,24	0,15	0,39	-2,00	0,08	0,23	-0,76	0,64	1,00
**Docosahexaenoic_acid**	-0,59	0,47	0,61	-0,80	0,39	0,51	-0,20	0,85	1,00
**Docosapentaenoic_acid**	-2,23	**0,05**	0,29	-4,19	**0,03**	0,22	-1,96	0,67	1,00
**Erucic_acid**	-0,39	0,65	0,77	-1,54	0,25	0,36	-1,15	0,43	1,00
**Icosadienoic_acid**	-0,77	0,35	0,52	-1,66	0,16	0,29	-0,89	0,54	1,00
**Eicosapentaenoic_acid**	-1,45	**0,05**	0,29	-1,99	**0,03**	0,22	-0,54	0,70	1,00
**Icosatrienoic_acid**	-2,01	0,10	0,33	-3,88	0,06	0,23	-1,87	0,67	1,00
**Icosenoic_acid**	-1,17	0,19	0,40	-2,21	0,09	0,23	-1,04	0,57	1,00
**Lignoceric_acid**	0,35	0,71	0,82	0,89	0,29	0,40	0,54	0,44	1,00
**Linoleic_acid**	-1,43	0,19	0,40	-2,28	0,12	0,28	-0,85	0,72	1,00
**Nervonic_acid**	-0,87	0,34	0,52	-2,57	0,11	0,26	-1,70	0,42	1,00
**Oleic_acid**	-1,66	0,14	0,39	-2,92	0,08	0,23	-1,26	0,67	1,00
**Tetrahydro-3,4-furandiol**	1,15	0,35	0,52	-1,57	0,62	0,72	-2,72	0,16	1,00
**A-Linolenic_acid**	-2,81	0,08	0,33	-3,80	0,08	0,23	-1,00	0,88	1,00
**Prostacyclin**	-2,47	**0,02**	0,29	-3,14	**0,02**	0,22	-0,67	0,83	1,00
**Palmitic_acid**	-1,27	0,19	0,40	-1,56	0,16	0,29	-0,29	0,86	1,00
**Stearic_acid**	-0,59	0,40	0,57	-0,79	0,32	0,43	-0,20	0,82	1,00
**13-keto-9Z,11E-octadecadienoic_acid**	-1,08	0,20	0,41	-1,18	0,20	0,31	-0,10	0,94	1,00
**19-/20-hydroxyarachidonic_acid**	-1,83	**0,04**	0,29	-2,46	**0,03**	0,22	-0,63	0,75	1,00
**Cholesterol**	-1,99	0,09	0,33	-2,86	0,07	0,23	-0,87	0,78	1,00

14,15,19-/14,15,20-trihydroxy-5,8,11-eicosatri	-0,89	0,35	0,52	-0,70	0,46	0,57	0,19	0,87	1,00
11(12)oxido-5,8,14-eicosatrienoic_acid	-1,83	**0,04**	0,29	-2,46	**0,03**	0,22	-0,63	0,75	1,00
14,15-dihydroxy-5,-8,11-eicosatrienoic_acid	-1,64	0,15	0,39	-1,64	0,17	0,29	0,01	1,00	1,00
19-oxoarachidonic_acid	-1,03	0,15	0,39	-0,76	0,26	0,36	0,27	0,76	1,00
9-keto-10E,12Z-octadecadienoic_acid	-1,15	0,18	0,40	-1,22	0,19	0,30	-0,07	0,96	1,00
Cholestanetriol_(5_alpha-cholestane-3_beta,_5,	-0,20	0,80	0,88	0,19	0,79	0,82	0,40	0,59	1,00
14(15)oxido-5,8,11-eicosatrienoic_acid	-1,83	**0,04**	0,29	-2,46	**0,03**	0,22	-0,63	0,75	1,00
Cholesterol_alpha-epoxide_(5,_6_alpha-epoxy-5-	-0,11	0,87	0,90	0,07	0,91	0,93	0,19	0,77	1,00
11,12,19-/11,12,20-trihydroxy-5,8,14-eicosatri	-0,89	0,36	0,52	-0,71	0,46	0,57	0,19	0,88	1,00
5(6)oxido-8,11,14-eicosatrienoic_acid	-1,90	**0,04**	0,29	-2,46	**0,03**	0,22	-0,56	0,78	1,00
Cis-9,10-epoxyoctadecanoic_acid_(cis-EODA)	-0,34	0,62	0,77	-0,30	0,68	0,77	0,04	0,95	1,00
SM_(D34:0)	-0,17	0,83	0,90	-2,57	0,16	0,29	-2,40	0,20	1,00
SM_(16:0)	-0,89	0,35	0,52	-3,99	0,09	0,23	-3,09	0,34	1,00
PC_(30:3)	-0,36	0,52	0,66	-0,37	0,53	0,64	-0,01	0,99	1,00
PC_(30:0)	-0,92	0,30	0,52	-3,63	0,07	0,23	-2,72	0,32	1,00
PC_(38:5)	-0,21	0,72	0,82	-0,19	0,76	0,82	0,02	0,97	1,00
PC_(38:4)	-0,45	0,46	0,61	-0,30	0,62	0,72	0,15	0,82	1,00
PC_(32:1)	0,83	0,33	0,52	1,14	0,17	0,29	0,31	0,59	1,00
PC_(36:1)	-0,63	0,47	0,61	-0,22	0,79	0,82	0,42	0,66	1,00
PC_(32:0)	0,14	0,86	0,90	0,82	0,23	0,35	0,68	0,27	1,00
PC_(36:2)	0,64	0,97	0,97	0,87	0,13	0,28	0,23	0,17	1,00
TAG_(54:5)	-3,60	0,13	0,39	-4,67	0,14	0,29	-1,08	0,94	1,00
PC_(34:6)	0,72	0,35	0,52	1,00	0,18	0,29	0,28	0,60	1,00
